# Utilization of immediate postpartum intrauterine device and its associated factors among women who gave birth in public hospitals in West Wollega Zone, Oromia, Ethiopia

**DOI:** 10.3389/fmed.2023.1238496

**Published:** 2023-11-23

**Authors:** Ararso Hordofa Guye, Efa Bayissa Kanea, Tadesse Nigussie, Derara Girma, Dame Banti Shambi

**Affiliations:** Department of Public Health, College of Health Sciences, Salale University, Fiche, Ethiopia

**Keywords:** intrauterine device utilization, postpartum family planning, post-partum intrauterine device, postpartum contraceptive, West Wollega Zone, Ethiopia

## Abstract

**Background:**

The utilization of an immediate postpartum intrauterine device (IPPIUD) during the postpartum period helps women to realize their desire for birth spacing and prevent unplanned pregnancies. However, many postpartum mothers do not undergo immediate postpartum family planning in developing countries, including Ethiopia, which consequently increases the risk of unplanned pregnancies and pregnancy-related complications.

**Objective:**

To assess the utilization of an IPPIUD and its associated factors among women who gave birth in public hospitals in the West Wollega Zone in 2022.

**Methods:**

An institutional-based cross-sectional study was conducted among 605 postpartum mothers who delivered their baby between 15 April and 15 May 2022 at public hospitals in the West Wollega Zone, Ethiopia. A systematic random sampling technique was used to select study subjects, and the data were collected using an interviewer-administered structured questionnaire, which was then entered into EpiData Entry version 4.6 and exported to the Statistical Package for Social Science version 26 for analysis. The variable with a *p*-value of ≤ 0.05 with an adjusted odds ratio and 95% confidence intervals was used to declare statistically significant association.

**Result:**

The prevalence of the utilization of the IPPIUD among respondents who gave birth in West Wollega public hospitals within 48 h was 27.2% (95% CI, 23.7–30.9). Age ranging between 25 and 34 years (AOR = 4.27, 95%CI:1.68–10.85), early initiation of antenatal care (ANC; AOR = 1.91, 95%CI: 2.8–10.01), adequate knowledge of IPPIUD (AOR = 4.71, 95%CI: 2.63–6.63), favorable attitude toward family planning (AOR = 3.35, 95%CI: 2.07–5.44), planning of pregnancy (AOR = 2.21, 95%CI: 1.37–4.11), and counseling (AOR = 4.14, 95%CI: 2.60–6.68) were factors that were significantly associated with the utilization of IPPIUD.

**Conclusion:**

According to the 2019 Ethiopia Mini Demographic and Health Survey (mini EDHS 2019), the utilization of an immediate postpartum intrauterine device was low, that is, 35%. Age of respondents, early initiation of antenatal care, favorable attitude toward, planning of pregnancy, adequate knowledge of, and counseling on IPPIUD utilization were significantly associated with the mother’s utilization of immediate postpartum intrauterine device. Thus, the zonal health office and health professionals should work toward encouraging all the women who gave birth at public hospitals to the utilization of immediate postpartum intrauterine devices by improving awareness among the women in that specific zone through counseling to increase the uptake of IPPIUD.

## Introduction

An immediate postpartum intrauterine device (IPPIUD) is a postpartum contraceptive inserted into a woman’s uterus after delivery of the placenta. It is made up of plastic copper intrauterine device type T380A (CU T-380A) and can be classified into post-placental, immediate postpartum, early postpartum, and extended postpartum intrauterine devices that are inserted within 10 min, 48 h, 48 h to 6 weeks, and 6 weeks to 1 year after birth, respectively (1–3). Postpartum family planning helps women to realize their desire for birth spacing and prevents them from having unplanned and closely spaced pregnancies throughout the first 12 months following childbirth (4). The immediate postpartum intrauterine device (IPPIUD) is safe and effective, with a low expulsion rate when compared to the interval intrauterine devices that are used to avoid unwanted pregnancies (5, 6), and is a highly convenient option for postpartum mothers who want a long-acting, reversible, non-hormonal protection from pregnancy that can be initiated during the critical postpartum period (7, 8).

Globally, in 2020, among the 1.9 billion women of reproductive age group, 57.19% of them had the desire to use family planning, 17% of them had utilized intrauterine devices, and 10% of did not use any contraceptive method (3, 9), and in Eastern and South-Eastern Asia, 18.6% of women utilized IPPIUD in order to avoid pregnancy (10). Family planning (FP) is recognized as a key life-saving intervention for mothers and their children (11), and the IPPIUD can promote the health of women and children by preventing unwanted pregnancies and financial, psychological, obstetric, and other health and health-related complications associated with closely spaced pregnancies, and its insertion does not require repeated healthcare visits for contraceptive refills (12–15).

In Africa, the utilization of IPPIUD is still low; the percentage of mothers who used an immediate postpartum intrauterine device within 48 h of placenta delivery was 4, 3.4, 1.1, and 0.3% in Rwanda, Zambia, Kenya, and Eretria, respectively (10, 16). In Ethiopia, the utilization of IPPIUD remains very low beside the high level of unmet need for postpartum family planning (3), and the report of the Maternal and Child Survival Program (MCSP) project showed that 8.55% of mothers had utilized an immediate postpartum intrauterine device within 48 h of delivery (4). A study conducted in the Bale Zone and the EngenderHealth report of the project of western Oromia revealed that the immediate postpartum intrauterine device was utilized by 12.4 and 8% of women, respectively (9, 17). Immediate postpartum is a critical period for the uptake of family planning to prevent unwanted pregnancy (18). However, given the high emphasis on contraceptive use, contraceptive use by mothers during this period is low (5, 6, 19).

Ethiopia has introduced IPPIUD initiatives to increase access to postpartum family planning and strengthen service provision, responding to women’s high unmet need for postpartum family planning (18). The Maternal and Child Survival Program (MCSP) initiated and introduced the postpartum family planning program in Ethiopia (18, 20), continued to extend its support to increase the availability of long-acting family planning and scale-up postpartum family planning (PPFP) at the national and facility levels, integrated IPPIUD services into national and reproductive health policies, trained maternity staff on its insertion and on counseling the mothers, provided on-site mentorship of newly trained healthcare providers, engaged experts in the fields, and advocated its benefits to policymakers (5, 21–23).

Previous studies have reported that the utilization of IPPIUD is significantly associated with sociodemographic characteristics such as age, marital status, educational status, occupation, and residential area and reproductive characteristics such as age at marriage, age at first delivery, number of pregnancies and births, number of antenatal care (ANC) follow-ups, and number of alive children (1, 5, 6, 19, 24, 25). However, notable actions were not taken at the household, community, and facility levels to maximize the possibility of making couples aware of all possible postpartum family planning options available to them, including immediate postpartum intrauterine device, to improve poor communication or a lack of communication between couples, and to encourage women to engage their male partners in postpartum family planning decisions (18, 25). Postpartum women are among those with the greatest unmet need for postpartum family planning (26), and however, they often do not receive the services they need to realize their desire for spacing births and reduce unwanted pregnancy and its consequences (27–29).

Moreover, there is limited evidence and information regarding immediate postpartum intrauterine device utilization, and studies conducted in Ethiopia did not address factors that were important in other African countries such as the number of ANC visits and pregnancy intervals of more than 2 years (29, 30). In general, the primary goal of this study was to provide current information related to IPPIUD utilization in the West Wollega Zone area for mothers, the community, and healthcare providers. Thus, the study was aimed to assess the utilization of immediate postpartum intrauterine device and the factors that affect the utilization of IPPIUD during the postpartum period for the use of IPPIUD among mothers who gave birth in public hospitals in West Wollega Zone, Oromia, Ethiopia.

## Materials and methods

### Study design, period, and area

**Figure 1:**
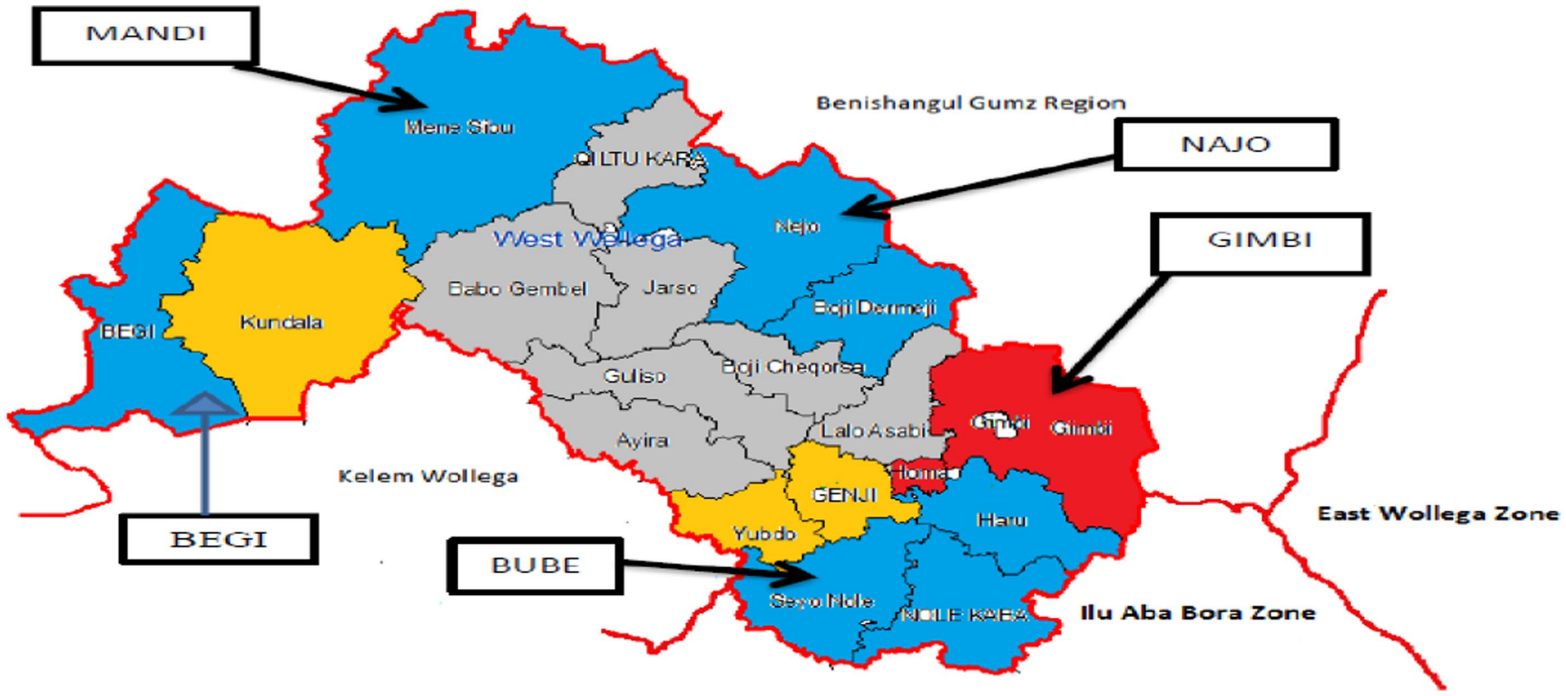
Study area map for the study of the immediate postpartum intrauterine device utilization and its associated factors among women who gave birth in hospitals in the West Wollega Zone, Oromia, Ethiopia, 2022.

The study design was an institution-based cross-sectional design and was conducted among women who gave birth at any one of the five public hospitals in the West Wollega Zone from 15 April to 15 May 2022. The West Wollega Zone is one of the 21 zones of the Oromia National Regional State, and Gimbi Town is the zonal capital city, which is located at a distance of 441 km to the west of Addis Ababa (Figure 1). Based on the 2007 census conducted by the Central Statistical Agency of Ethiopia (CSA), this zone has a total population of approximately 1,741,567 (men = 877,290, women = 864,277) individuals and a total land area of 1,274,501 ha (24). Different governmental and non-governmental health institutions are providing healthcare services for the community, including 5 public hospitals, 2 private hospitals, 67 health centers, 488 health posts, 2 specialty clinics, 22 medium clinics, 212 primary clinics, as well as different private clinics, and drug stores.

The total number of health professionals in the zone is 2,806, among whom there are 60 physicians, 147 health officers, 612 nurses, 257 midwives, 387 pharmacy professionals, 223 laboratory professionals, and 1,120 other professions, including health extension workers (24). The average number of mothers who gave birth in all study area hospitals by both spontaneous vaginal delivery (SVD) and cesarean section (CS) in the previous 3 months before the start of the study were identified as follows: 368 in Gimbi General Hospital, 302 in Najo General Hospital, 250 in Mendi Primary Hospital, 184 in Begi Primary Hospital, and 174 in Bube Primary Hospital.

Generally, major services provided at all hospitals related to Maternal and Child Health (MCH) services are family planning, ANC, and delivery services, and all public hospitals provide these services for free. In addition, all modern contraceptive techniques such as long-acting contraception such as IUCD are available in all hospitals (Source: West Wollega Health Office).

#### Source population and study population

All women who gave birth in public hospitals in the West Wollega Zone were the source population, and the selected women who gave birth in public hospitals in the West Wollega Zone during the study period were the study population.

### Eligibility criteria

Women who gave birth in public hospitals in the West Wollega Zone during the study period and those who fulfilled the eligibility criteria for IUCDs were included in the study. Women who were sick, unable to respond, who had active sexually transmitted diseases, who had ruptured membranes for more than 24 h, who had a ruptured uterus, unresolved postpartum hemorrhage, or uterine tony, and who met the following WHO exclusion criteria were excluded from the study. The exclusion criteria also included those women who showed evidence of puerperal infections such as temperatures (T) >38°C and pulse rates (PR) >100 beats per minute, who were clinically unstable at the time of birth, who had a history of complications during the intrapartum period, who had allergies to the metals used in the CU T380A IUCD, who had stage 4 HIV/AIDS without ARV therapy, and who had opted out following enrolment.

### Sample size determination

The sample size was calculated by using Epi Info version 7.2.5 and Stat Calc software programs and determined by using the single population proportion formula with the assumptions of the proportion of women who utilized IPPIUD from the previous study, which was 35.6% (*p* = 0.356) (31), with a 95% confidence level (CL) of 1.96, a margin of error of 4%, and a 10% non-response rate.

Also, the sample size was calculated for the second objective by using EPI-Info version 7.2 and Stat Calc software programs using the double population proportions formula by considering various factors that were significantly associated with the outcome variables using the following assumptions of a 95% CL and 80% power, where a one-to-one ratio was considered. Then, by comparing the first and second objectives, the factor that gives the maximum sample size was considered and the maximum calculated sample size was 550 for the variable “heard about IPPIUD.” After adding a 10% non-response rate, the final sample size was 605.

### Sampling procedure

In this study, all five public hospitals in the West Wollega Zone that provide IPPIUD services were selected. The sample size for each hospital was allocated based on the average monthly delivery flow of one quarter. The total population served by those hospitals was 1,278 (Gimbi General Hospital = 368, Mendi Primary Hospital = 250, Nejo General Hospital = 302, Begi Primary Hospital = 184, and Bube Primary Hospital = 174) (Table 1).

**Table 1:** Proportional allocation of the study population to the respective public hospitals in the West Wollega Zone, Oromia Region, Ethiopia in the year 2022 (*n* = 605).

Hospitals	Population	Proportional estimation	Sample taken
Bube	174	174 × 605/1,278	82
Gimbi	368	368 × 605/1,278	174
Najo	302	302 × 605/1,278	143
Mendi	250	250 × 605/1,278	118
Begi	184	184 × 605/1,278	88
Total	1,278	1278 × 605/1,278	605

**Figure 2:**
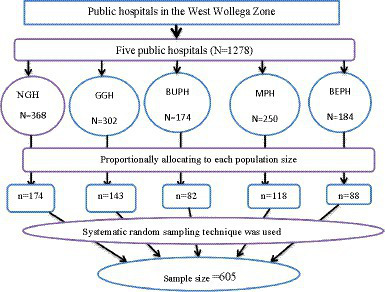
Schematic representation of systematic random sampling techniques for the study that was conducted in public hospitals in the West Wollega zone, Oromia, Ethiopia in the year 2022. BPH, Begi Primary Hospital; NGH, Nejo General Hospital; MPH, Mendi Primary Hospital; GGH, Gimbi General Hospital; BPH, Bube Primary Hospital.

The sample of 605 participants was obtained by proportionally allocating those five hospitals by considering their monthly delivery flows for one quarter. Consequently, study subjects were recruited by a systematic random sampling technique at every K interval of 2 (K=N/n = 1278/605 = 2), so every woman who gave birth in the hospital was recruited as a study subject in each hospital until the total sample size for this study was obtained (Figure 2).

## Study variables

Immediate postpartum intrauterine device utilization is the dependent variable and the independent variables are as follows: *sociodemographic characteristics*: age of the mother, marital status, educational status of women, husband’s educational status, occupation of mothers, husband’s occupation, mother’s residential area, with whom mothers live, family size, and distance from hospitals; *reproductive health history of mothers on IPPIUD*: age at marriage, age at first delivery, number of pregnancies, number of deliveries, whether current pregnancy was planned, number of births, delivery pregnancy, current birth outcome, weeks at ANC initiation, number of ANC follow-ups, number of alive children, desire for future children, and place of delivery; *knowledge of mothers on IPPUCD*: prevents pregnancy for more than 10 years, can be inserted immediately after delivery, used by breastfeeding mothers, causes changes in the menstrual bleeding pattern, does not cause cancer, and can be removed at any time; *the attitude of mothers on PPIUD*: invades privacy during insertion, restricts normal activity, causes damage to the uterus, feeling of moving through the body after insertion, causes severe pain during insertion and removal, feeling of interference during sexual intercourse, and impairing future fertility.

### Measurements and operational definitions

#### Utilization of IPPIUCD

Women who gave their verbal consent to use and who already had inserted in them the intrauterine device after the post-placental period within 10 min to 48 h of delivery (4, 5).

#### Knowledge of mothers on IPPIUCD

Women were asked 10 knowledge questions about the utilization of IPPIUCD and the correct response was scored 1 and the incorrect one was scored 0. After computing the sum score for each respondent, the mean score was calculated, and those who scored greater than the mean were considered as having “adequate knowledge” and those who scored below the mean as having “inadequate knowledge” about the utilization of IPPIUCD (19, 28, 32).

#### Attitude toward IPPIUCD

Eight questions were asked to assess the attitude of women in IPPIUCD using the Likert Scale (1 = strongly disagree, 2 = disagree, 3 = neutral, 4 = agree, and 5 = strongly agree). After computing the sum score for each respondent, the mean score was calculated and those who scored above the mean were considered as having a “favorable attitude” and those who scored below the mean as having an “unfavorable attitude” toward the utilization of IPPIUCD (28, 32).

### Data collection tool and procedures

Data were collected through face-to-face interviews using structured questionnaires that were adapted by reviewing different literatures (4, 5, 22, 33). The questionnaire was prepared in English and translated to Afan Oromo by a language expert to better understand both the data collectors and the respondents and then translated back to the English version to check the consistency. A reliability coefficient (Cronbach’s alpha) was calculated for Likert scale questions in SPSS, which is α = 0.914. The questionnaire has the following parts such as sociodemographic characteristics, reproductive and obstetric characteristics, knowledge of family planning and immediate postpartum intrauterine devices, the attitude of the mother toward IPPIUD, and utilization of immediate postpartum intrauterine devices.

The supervisors together with data collectors were assigned to each hospital. The data collectors were asked to fill out the questionnaires between immediately after delivery of the placenta and within 48 h of the postpartum period and submit them when they were finished. The supervisors organized the filled questionnaires from data collectors and submitted them to the principal investigator each day. Two diploma and three BSc midwives were recruited as data collectors and two BSc midwives as supervisors based on their language skills and supervision experience. There were familiarized with the study area and trained on the study objectives, the method of data collection, and the tools for data collection. Trained data collectors collected the data at all hospitals using a revised version of the data collection tool, and they interviewed the mothers who gave birth in any of the five study hospitals.

### Data quality assurance

To ensure the quality of the data, a step-by-step process was applied to minimize bias and errors during the study design, sampling, questionnaire development, data collection, and data processing. To ensure data quality, the data collection tool was developed in English, translated to the local language, Afan Oromo, and then translated back to English by a language expert (an individual who has good knowledge of both English and Afan Oromo languages) to ensure its consistency. A pretest of the questionnaire was conducted at Danbidollo Hospital, which is not included in the study, by taking 5% of the total sample size (30 participants) to assess the consistency and accuracy of the tool. After the pretest of the tools, the necessary modifications were made to the questionnaires before use for actual data collection.

Data collectors and supervisors were given a 2-day training on the study objectives, the method of data collection, and the tools for data collection. During data collection, the data collectors were supervised regularly, and necessary feedback was given. Information was checked for completeness and internal consistency before and during the data processing phase. Incomplete data were discarded. Questionnaires were checked to ensure all data had been filled in to avoid missing data. The investigator checked for the completeness and consistency of questionnaires filled out by the data collectors to ensure the quality of the data.

### Data processing and analysis

Filled-in questionnaires were checked for completeness and consistency. The data was coded and entered into Epi-data version 4.6 before being exported to SPSS version 26 for cleaning and analysis. After the gathered data were checked for missing values, outliers, and fulfillment of assumptions, the variables were computed and recoded by the transform function of SPSS. Descriptive statistics were summarized using frequencies, percentages, mean, standard deviation, and interquartile range and were presented in the form of figures, tables, and text.

A bivariable analysis was conducted for all independent variables against each dependent variable separately using binary logistic regression to see their association. Variables that showed a significant association in the bivariable analysis with a *p*-value of ≤ 0.25 and 95% CI were candidates for multivariable logistic regression and were entered into multiple logistic regression to identify their independent effects. A multivariable logistic regression analysis was conducted after checking the model fitness test using the Hosmer-Lemeshow goodness-of-fit test. The *p*-value of the Hosmer-Lemeshow goodness-of-fit test of the model was checked, and it was well fitted with *p* = 0.867, and the omnibus test was significant, and the assumption was fulfilled since the *p*-value was greater than 0.05.

Multicollinearity was also checked by standard error for the β-coefficient (a standard error > 2 indicates its presence as a cutoff point), and the backward logistic regression selection method was used to identify the variable remaining for the final. The variables with a *p*-value of ≤ 0.05 with a respective adjusted odds ratio (AOR) and 95% CIs were used to declare the significantly associated factors with immediate postpartum intrauterine device utilization.

## Results

### Sociodemographic characteristics of study respondents

A total of 599 respondents were interviewed with a response rate of 99% since six participants were not included in the study as a result of non-responses during data collection. The mean and standard deviation of the respondent’s age was 27.49 (6.51) years. Of the study respondents, 292 (48.7%) were in the age range of 25–34 years, 130 (21.9%) in the age range of 15–24 years, and 167 (29.4%) in the age range of 34–49 years. Out of the study respondents, the largest proportion, 544 (90.8%), were married. Of the study respondents, 135 (22.5%) had a college degree and above. Regarding the occupational status of women and their husbands, 315 (52.6%) and 193 (32.8%) were homemakers and farmers, respectively. The majority of the study respondents, i.e., 537 (89.6), were living with their husbands. Almost half of the study respondents, 305 (50.9%), were living in urban areas (Table 2).

**Table 2:** Sociodemographic characteristics of women who gave birth in public hospitals of West Wollega Zone in the year 2022 (*n* = 599).

Variables	Category	Frequency (%)
Age (completed years)	15–24	131 (21.9)
	25–34	292 (48.7)
	35–49	176 (29.4)
Area of residence	Rural	294 (49.1)
	Urban	305 (50.9)
Marital status	Single	13 (2.2)
	Married	544 (90.8)
	Divorced	29 (4.8)
	Widowed	13 (2.2)
Mothers’ education status	No formal education	185 (30.9)
	Primary education	146 (24.4)
	Secondary education	133 (22.2)
	College and above	135 (22.5)
Mothers’ occupational status	Housewife	315 (52.6)
	Government employee	133 (22.2)
	Private employee	71 (11.9)
	Merchants	66 (11)
	Others**	13 (2.3)
Husbands’ occupational status	Government employee	204 (34.7)
	Private employee	107 (18.2)
	Merchants	63 (10.7)
	Farmer	193 (32.8)
	Others***	21 (3.6)
Distance from the hospital (km)	≤3	193 (32.2)
	>3	406 (67.8)

** indicates the mother’s occupational status in a variable category with others as “Daily laborer, student.” The label *** indicates the husband’s occupational status in a variable category with others as “Daily laborer, student.” Therefore, since the occupational category with others for mothers and husbands is similar we have agreed to use the label ** for booth groups as “Others **= Daily laborer, student”.

### Reproductive characteristics of the study respondents

The mean and standard deviation of marriage age were 19.28 (2.58) years. Four hundred eighty-two (81.7%) had married at an age greater than or equal to 18 years. The of age 566 (94.5%) mothers at first delivery was 18 years or above. Two hundred eighty-six (54.6%) women had given birth two to four times, and 53 (8.8%) women had given birth five or more times. The mean (SD) number of alive and wanted children in life was 2.46 (1.76) and 4.14 (1.57), respectively. Five hundred twenty-seven (88.0%) mothers had antenatal care follow-up for their then-current pregnancy. One hundred thirty-nine (23.2%) had initiated antenatal care services at less than or equal to 16 weeks for their then-current pregnancy. Four hundred ninety-four (82.5%) mothers had used family planning before this pregnancy. Two hundred thirty-eighty (45.2%) and two hundred fifty-eight (49%) women had made 2–3 and 4 and above ANC visits, respectively. Most mothers, 430 (71.8%), had discussed family planning with their husbands. Approximately 543 (90.7%) women responded that both husbands and wife had decided on the number of children they wanted to have together (Table 3).

**Table 3:** Reproductive characteristics of mothers who gave birth in public hospitals of West Wollega Zone, Oromia, Ethiopia (*n* = 599).

Variables	Category	Frequency (%)
Number of pregnancy	1st pregnancy	187 (31.2)
	2nd–4th pregnancy	334 (55.7)
	≥5th pregnancy	78 (13.1)
Antenatal care follow-up ≤16 weeks	No	460 (76.8)
	Yes	139 (23.2)
Interpregnancy interval	≤2 years	200 (47.7)
	>2 years	219 (52.3)
Mode of current delivery	Spontaneous delivery	371 (58.3)
	Breach delivery	70 (8.3)
	Vacuum delivery	158 (23.0)
The outcome of the current birth	Alive	594 (99.2)
	Dead	5 (0.8)
whether their then-current pregnancy was planned	No	107 (17.9)
	Yes	492 (82.1)
number of children that you want to have in your life	≤3	187 (31.2)
	≥4	412 (68.8)
Want to have a child within 2 years	No	102 (17.0)
	Yes	497 (83.0)
Deciding on the number of children you want to have	Respondent only	22 (3.7)
	Husband only	33 (5.51)
	Both of them	543 (90.7)

### Family planning and types of family planning used before this pregnancy by the mothers

**Figure 3:**
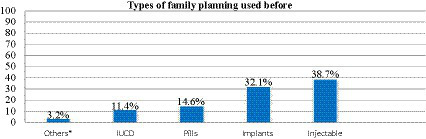
Types of family planning used by respondents before their then-current pregnancy in public hospitals in West Wollega Zone in the year 2022. * Condoms and emergency pills.

A total of 494 respondents (82.5%) had used the family planning method before their then-current birth. The majority of mothers (38.7%) had used injectable contraceptives (Figure 3).

### Knowledge of mothers on immediate postpartum intrauterine device

The mean score of the knowledge status of the respondents was 4.32 (±3.35 SD). The study reported that 316 (52.8%) respondents had inadequate knowledge and 283 (47.2%) had adequate knowledge of immediate postpartum intrauterine devices. In total, 252 (58.8%) participants knew that an IUD can prevent pregnancies for more than 10 years, 234 (39.1%) participants knew that an IUD can be inserted immediately after delivery, 259 (43.2%) knew that an IUD is immediately reversible; and 202 (33.7%) knew that an IUD does not cause cancer (Table 4).

**Table 4:** Knowledge of the respondents on immediate postpartum intrauterine device utilization among mothers who gave birth in public hospitals in West Wollega Zone, Oromia, Ethiopia in the year 2022 (*n* = 599).

Variables	Category	Frequency (%)
Can prevent pregnancy for more than 10 years	No	247 (41.2)
	Yes	352 (58.8)
Can be inserted immediately after delivery	No	365 (60.9)
	Yes	234 (39.1)
Has no interference with sexual intercourse	No	409 (68.3)
	Yes	190 (31.7)
Is immediately reversible	No	340 (56.8)
	Yes	259 (43.2)
Does not cause cancer	No	397 (66.3)
	Yes	202 (33.7)
Can be used by breastfeeding mothers	No	277 (46.2)
	Yes	322 (53.8)
May cause changes in the bleeding pattern	No	362 (60.4)
	Yes	237 (39.6)
Can be used by HIV-positive mothers who adhere to treatment	No	410 (68.4)
	Yes	189 (31.6)
Given free of charge in the delivered hospital	No	264 (44.1)
	Yes	335 (55.9)
Can be removed any time you want	No	330 (55.1)
	Yes	269 (44.9)

### Mother’s attitude toward IPPIUD utilization

The mean score (±SD) value of the participant’s attitude toward the immediate PPIUD utilization were 20.79 (SD ± 6.65). Using the sum score as the cutoff point, the study showed that 184 (30.7%) mothers had a favorable attitude toward the utilization of immediate postpartum intrauterine devices. In total, 199 (33.2%) mothers disagreed that IUD insertion inside the uterus does not lead to a loss of privacy, while 38 (6.3%) strongly agreed to the loss of privacy. Two hundred fifteen (35.9%) participants agreed that using IPPIUD does not restrict normal activities. Additionally, 192 (32.1%) agreed that IPPIUD does not move through the body after insertion and 259 (26.5%) agreed and 96 (16%) highly agreed that IPPIUD does not interfere with sexual intercourse. Also, 88 (14.7%) participants highly disagreed with IPPIUD’s ability to harm a woman’s uterus (Table 5).

**Table 5:** Attitude of the mothers toward immediate postpartum intrauterine device utilization among women who gave birth in public hospitals in West Wollega Zone, Oromia, Ethiopia in the year 2022 (*n* = 599).

Variables	Category *n* (%)				
	Strongly disagree	Disagree	Neutral	Agree	Strongly agree
Inserting IPPIUD inside the uterus leads to a loss of privacy	147 (24.5)	199 (33.2)	171 (28.5)	44 (7.3)	38 (6.3)
IPPIUD does restrict normal activities	112 (18.7)	215 (35.9)	198 (33.1)	60 (10.0)	14 (2.3)
IPPIUD moves through the body after insertion	112 (18.7)	192 (32.1)	215 (35.9)	62 (10.4)	18 (3.0)
IPPIUD can interfere with sexual intercourse	96 (16.0)	159 (26.5)	248 (41.4)	74 (12.4)	22 (3.7)
IPPIUD harms a woman’s uterus	88 (14.7)	169 (28.2)	232 (38.7)	78 (13.0)	32 (5.3)
Insertion and removal of IUD did cause extensive pain	69 (11.5)	164 (27.4)	205 (34.2)	117 (19.5)	44 (0.3)
Using IPPIUD can cause irregular bleeding	82 (13.7)	131 (21.9)	232 (38.7)	116 (19.4)	38 (6.3)
Using IPPIUD impairs future fertility	97 (16.2)	97 (16.2)	97 (16.2)	97 (16.2)	97 (16.2)

### Immediate postpartum intrauterine device utilization and the reasons for utilization

The study showed that the prevalence of utilization of immediate postpartum intrauterine devices was 27.2% (95%CI: 23.7–30.9). A total of 111 (52.6%) respondents were counseled for immediate postpartum intrauterine devices. During ANC visits, 53 (32.5%) and 44 (27.0%) mothers had information about immediate postpartum IUD and needed a long-acting, safe, and effective method to use the immediate postpartum IUD, respectively (Table 6).

**Table 6:** Reasons for immediate postpartum intrauterine device utilization among mothers who gave birth in public hospitals in West Wollega Zone, Oromia, Ethiopia in the year 2022.

Variables	Category	Frequency (%)
Utilization of IPPIUD after delivery	Had information on IPPIUD during ANC visits	53 (32.5)
	Husband’s and relatives’ opinions	17 (10.4)
	Recommended by the service provider	14 (8.6)
	Had side effects from other family planning use	30 (18.4)
	Needed a long-acting, safe, and effective method	44 (27.0)
	Recommended by peers	5 (3.1)
Time counseled for IPPIUD	During antenatal period	111 (52.6)
	On admission while in labor	26 (12.3)
	Immediately after delivery	74 (35.1)

### The reasons for respondents not utilizing an immediate postpartum intrauterine device

**Figure 4:**
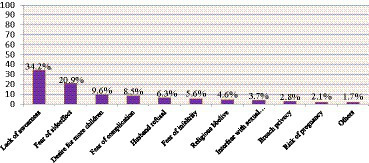
The reasons respondents did not utilize immediate postpartum intrauterine devices among women who gave birth in public hospitals in West Wollega Zone, Oromia, Ethiopia in the year 2022. Others: peers or relatives.

The most common reasons mentioned by study respondents to not utilize an immediate postpartum intrauterine device were a lack of awareness for 204 (34.2%) respondents followed by a fear of side effects for 125 (20.9%) respondents (Figure 4).

### Factors associated with immediate postpartum intrauterine device utilization

A binary logistic regression was performed to assess the association of each independent variable with immediate postpartum intrauterine device utilization. Of all the assessed factors, 14 variables showed association in the bivariable analysis: age of respondents; educational status of mothers; age at first delivery; antenatal care initiation at or less than 16 weeks; status of pregnancy (planned or not); family planning used before this birth; want to have a child within 2 years; knowledge status; ANC follow-up during current pregnancy; number of children you want to have in your life; discussing with their partner; attitude status; decided on the number of children you want to have; and counseled on immediate postpartum intrauterine device.

The variables with a *p*-value of less than 0.25 were entered into the multivariable logistic regression model after controlling for confounders. The results of the multivariable logistic regression model revealed that age of the respondents, antenatal care initiation at or less than 16 weeks, mothers whose current pregnancy is planned, adequate knowledge of immediate postpartum intrauterine devices, favorable attitude, and mothers who had been counseled on IPPIUD were found to be significantly associated with immediate postpartum intrauterine device utilization at a *p*-value of ≤ 0.05 together with 95% confidence intervals.

According to this study, age of the respondents were associated with immediate postpartum intrauterine device utilization: Mothers in the age groups of 25–34 years (AOR = 2.45, 95% CIs: 1.32–4.89) and 35–49 years (AOR = 1.37, 95% CIs: 1.02–2.81) were 2.45 times and 1.37 times more likely to utilize IPPIUD as compared to those in the age group of 15–24 years, respectively.

The study revealed that antenatal care initiation at or less than 16 weeks was significantly associated with immediate postpartum intrauterine device utilization. The mothers who had started antenatal care visits at an early gestational age were almost two times more likely to utilize IPPIUD than those who started later (AOR = 2.25, 95% CIs: 1.35–3.76). The results of the study revealed that those mothers whose current pregnancy was planned were two times more likely to use immediate postpartum intrauterine devices than mothers whose current pregnancy was unplanned (AOR = 2.21, 95%CIs: 1.37–4.11).

The study showed that mothers who had adequate knowledge of immediate postpartum intrauterine devices after delivery were 4.71 times more likely to use them than their counterparts (AOR = 4.71, 95% CIs: 2.63–6.63). Mothers who had favorable attitudes were three times more likely to utilize immediate postpartum intrauterine devices than mothers who had unfavorable attitudes (AOR = 3.35, 95% CIs: 2.07–5.44). Mothers who had been counseled on IPPIUD were four times more likely to utilize IPPIUD than those who had not been counseled (AOR = 4.14, 95% CIs: 2.60–6.68) (Table 7).

**Table 7:** Factors associated with immediate postpartum intrauterine device utilization among mothers who gave birth in hospitals in West Wollega zone, Oromia, West Ethiopia, 2022 (*n* = 599).

Variables category		Utilization of IPPIUD		COR 95%CIs	AOR 95%CIs
		Yes (%)	No (%)		
Age of respondents	15–24 years	21 (3.5)	110 (18.4)	1	1
	25–34 years	96 (16.0)	196 (32.7)	2.57 (1.52–4.35)	2.45 (1.32–4.89)*
	35–49 years	46 (7.7)	130 (21.7)	1.85 (1.04–3.30)	1.37 (1.02–2.81)*
Mothers’ education level	No formal education	41 (6.8)	144 (24.0)	1	1
	Primary education (1–8 K)	42 (7.7)	104 (17.4)	1.42 (0.86–2.34)	0.99 (0.53–1.86)
	Secondary education (9–12 K)	38 (6.3)	95 (15.9)	1.41 (0.84–2.34)	1.35 (0.69–2.62)
	College and above	42 (7.0)	93 (15.5)	1.59 (0.96–2.62)	1.18 (0.62–2.27)
Age at first delivery	<18	4 (0.7)	29 (4.8)	1	1
	≥18	159 (26.5)	407 (67.9)	2.83 (0.98–8.18)	0.52 (0.1–1.99)
ANC follow-up during the then-current pregnancy	Yes	156 (26.0)	377 (62.9)	3.49 (1.56–7.81)	0.89 (0.23–3.44)
	No	7 (1.2)	59 (9.8)	1	1
Weeks started at ANC follow-up	≤16	54 (9)	85 (14.2)	2.05 (1.37–3.06)	2.25 (1.35–3.76)*
	>16	109 (18.2)	351 (58.6)	1	1
Whether pregnancy was planned	Yes	138 (23)	306 (51.1)	2.35 (1.46–3.76)	2.21 (1.37–4.11)*
	No	25 (4.2)	130 (21.7)	1	1
Number of children you want to have in your life	≤3	57 (9.5)	130 (21.7)	1.27 (0.86–1.85)	1.08 (0.77–2.11)
	≥4	106 (17.7)	306 (51.1)	1	1
Want to have a child within 2 years	Yes	128 (21.4)	369 (61.6)	1	1
	No	35 (5.8)	67 (11.2)	1.53 (0.95–2.37)	1.31 (0.7–2.35)
Used family planning before the then-current birth	Yes	35 (5.8)	61(10.2)	1.68 (1.06–2.67)	1.11 (0.62–1.99)
	No	128 (21.4)	375 (62.6)	1	1
Decided the number of children you want	Yes	129 (21.5)	296 (49.4)	1.79 (1.17–2.75)	1.56 (0.89–2.72)
	No	34 (5.7)	140 (23.4)	1	1
Knowledge of respondents	Inadequate knowledge	41 (6.8)	275 (45.9)	1	1
	Adequate knowledge	122 (20.4)	161 (26.9)	5.08 (3.39–7.61)	4.71 (2.63–6.63)**
Attitude of respondents	Unfavorable	78 (13)	337 (56.3)	1	1
	Favorable	85 (14.2)	99 (16.5)	3.71 (2.5.43)	3.35 (2.07–5.44)**
Counseled on IPPIUD	No	76 (12.7)	361 (60.3)	1	1
	Yes	87 (14.5)	75 (12.5)	5.51 (3.71–8.18)	4.14 (2.60–6.68)**
Partner discussion on family planning	No	99 (16.5)	298 (49.7)	1	1
	Yes	64 (10.7)	138 (23)	1.39 (0.96–2.03)	1.2 (0.74–1.95)

*Statistically significant at a *p*-value of < 0.05, **p-value < 0.001, 1 = Reference, COR, crude odds ratio; AOR, adjusted odds ratio. We have used the bold values in table 7 to show the statistically significant category of the variables with adjusted odds ratio (AOR) and 95 % confidence interval.

## Discussion

This study aimed to assess the prevalence of immediate postpartum intrauterine contraceptive device utilization and identify factors that affect its utilization. The prevalence of immediate postpartum intrauterine contraceptive device utilization within 48 h of delivery was 27.2% (95% CIs: 23.5–30.7). This finding was in line with the studies conducted in public hospitals in Addis Ababa with a prevalence of 26.6% (3), Debre Berhan of 25.4% (5), Gondar of 48.4%, northwest Ethiopia (8), and Bahir Dar of 44% (34) and public health facilities in Gamo Zone of 26.4% (35).

The possible reasons for this similarity might be the level of awareness, early initiation of antenatal care of respondents, adequate knowledge of, and favorable attitude toward IPPIUD in the study areas (31). This finding was also in line with the studies conducted in Rwanda, Malaya of Cameroon, and India with the prevalence of 28.1% (30), 28.4% (16), and 28.33% (10). The possible reason for this similarity might be that women who utilized IPPIUD discuss with unutilized women its utilization benefits, the procedures, and the time taken, which might decrease the fear of women toward IPPIUD utilization.

The finding of the present study regarding the prevalence of IPPIUD utilization was higher than the studies conducted in Bale Zone (12.4%) (17); in Jimma University Medical Center (10.5%) (33); in Dessie Town (7%) (34); in Adama and Olenchiti (12.4 and 4.8% in the intervention and non-intervention groups, respectively) (11); at Saint Paul’s Millennium Medical College (7.8%) (23); and in Sidama Zone, south Ethiopia (21.9%) (4). These variations might be attributed to sociodemographic characteristics of the participants among study areas; differences in samples; time differences; misconceptions about intrauterine devices in the study areas; and to the existence of healthcare provider training and material support from a non-governmental organization in the study area.

The result of this finding was lower than found in a study conducted in Arsi Negele, which found that approximately 33.5% of study respondents had utilized IPPIUD (36), and a study conducted in Gamo Zone, southern Ethiopia, which found 35.6% of them had utilized IPPIUD (35). This inconsistency may be due to differences in the study design, sample size, and improvement of the health facilities on service provision of family planning with time. This result of this finding is also lower than that found in a study conducted in central India in a tertiary care center, in which it was reported that 36% of respondents had utilized IPPIUD (37), a study conducted in Minnesota on Somali immigrants, in which 39.7% of them had utilized IPPIUD (15), and a study conducted in Tanzania, in which 31.6% of them had utilized IPPIUD (38). These variations of immediate postpartum intrauterine device utilization might be attributed to sociodemographic characteristics variations due to the difference in the level of awareness and educational status of respondents, the difference in a sample, cultural beliefs, unfavorable attitudes, and misconceptions about intrauterine devices in the study areas (31, 35).

According to this study, those mothers in age category of 25–34 years and 34–49 years were more likely to utilize immediate postpartum intrauterine device compared to those within the age group of 15–24 years. The result of the finding is consistent with a study conducted in Gondar, Northwest Ethiopia (8). This might be due to older women being more willing to utilize immediate postpartum intrauterine devices than younger women since the age of the women is associated with increased awareness and understanding. It is well recognized that age plays an important role in women’s utilization of immediate postpartum intrauterine device and that maternal age may sometimes serve as a proxy for an accumulated understanding of long-acting family planning services (6). Age may be the factor that may have a positive influence on the accepting of IPPIUD utilization among women.

However, this finding was inconsistent with the study conducted in Debra Tabor (25) and Ambo Town, western Ethiopia (39). This inconsistency may be due to variations in respondents’ sociodemographic characteristics of the study area; the time gap between the study and sample size; the difference in awareness of intrauterine devices; the educational status of the study respondents; various misconceptions about IPPIUD in the study areas; differences in the study design; and variations in the health facilities’ provision of family planning services over time.

The results of this study revealed that those mothers who had planned their pregnancy were more likely to utilize immediate postpartum intrauterine devices than those mothers who had not planned their pregnancy. This finding is consistent with studies conducted in the Gamo Zone, southern Ethiopia (35); Debre Berhan, Ethiopia (5); and Addis Ababa, Ethiopia (23). This could be due to mothers’ understanding and attitude on timing and spacing of births by having a planned pregnancy through the utilization of immediate postpartum intrauterine devices after delivery.

The study also found that those mothers who had adequate knowledge of immediate postpartum intrauterine devices immediately after delivery were more likely to utilize IPPUD than their counterparts. This finding is consistent with studies conducted in Nigeria (40) and Rwanda (30), in Nepal (14), and in Durban, South Africa (28). The possible explanation for this finding is that as mothers get information on immediate postpartum intrauterine device utilization, their awareness of IUDs increases and their acceptance of the immediate postpartum intrauterine device improves.

The mothers who had favorable attitudes toward IPPIUD were more likely to utilize immediate postpartum intrauterine device than those mothers who had unfavorable attitudes. The current finding is in line with the studies conducted in Debre Berhan Town (5), Nigeria (40), and Tanzania (38). This may be due to women who have a favorable attitude toward immediate postpartum intrauterine device utilization having the self-initiative to know about the advantages and disadvantages of these devices.

The result of this study revealed that those mothers who were counseled on IPPIUD were more likely to utilize IPPIUD than those who had not been counseled. The study is consistent with a study conducted in Han Health Center, Bahir Dar, Ethiopia (6), a study conducted in Debre Berhan, Ethiopia (5), a study conducted in Kebri Beyan, Somali Region (25), and a study conducted in Jima University Medical Xenter (33) and a study conducted in the Sidama Zone (4). The possible explanation for this finding is that as mothers get counseling on immediate postpartum intrauterine device utilization at maternal, neonatal, and child health points of contact within the health system, their awareness about the importance of IUDs would be improved.

### Strengths and limitations of the study

Only those women who gave a birth at all five public hospitals that provides IPPIUD service in the West Wollega Zone were included in this study. As a limitation, social desirability bias may occur due to the interview time of the questions, i.e., immediate after delivery, on some variables during data collection, and since the study was conducted in public hospitals; women who gave birth at health centers, private health facilities, and at home were not included in the study, which is another limitation.

### Conclusion

Immediate postpartum intrauterine device utilization was low when compared to the national targets of Ethiopia predicted during the mini EDHS 2019, which was 35% (41). Age of respondents, initiation of antenatal care at or less than 16 weeks, pregnancy status of women as planned or not, knowledge status, attitude status, and counseling on the immediate postpartum intrauterine device were significantly associated with mothers’ utilization of the immediate postpartum intrauterine device.

### Recommendation

Health professionals should work toward encouraging all the women who gave birth at public hospitals to utilize an immediate postpartum intrauterine device through the integration of its services in routine maternal, neonatal, and child health service areas to increase the uptake of IPPIUD. Pregnant women should be given awareness about IPPIUD through counseling during their ANC appointments to provide them with a long-acting, safe, and effective protection against unwanted pregnancy, especially younger women. Furthermore, improving the knowledge and attitude of pregnant women toward IPPIUD utilization starting from ANC visit initiation to delivery is recommended to increase the prevalence of IPPIUD utilization.

## Data availability statement

The original contributions presented in the study are included in the article/supplementary material, further inquiries can be directed to the corresponding author.

## Ethics statement

Ethical approval was obtained from the ethical review committee of College of Health Science, Salale University, Fiche, Ethiopia. Permission letters were obtained from all public hospitals before conducting data collection, and the gathered data were secured at all levels. The data collectors explained the objective of the study to ensure the willingness of the study participants before filling in the questionnaire and informed the participants on the confidentiality of any information they provide. Informed written consent was obtained from each respondent after explaining the purpose and procedures. Considering the sensitivity of this research, all basic principles of human research ethics (respect of persons, beneficence, voluntary participation, confidentiality, and justice) have been secured.

## Author contributions

AHG, EBK, and TN participated in conceptualization, data curation, formal analysis, investigation, funding acquisition, methodology, project administration, resources, software, supervision, validation, visualization, writing original draft, writing review, and editing the manuscript. DG and DBS were involved in data curation, methodology, resources, analysis, investigation, supervision, validation, writing review, and editing the manuscript. All authors contributed to the article and approved the submitted version.

## Glossary

ANC: Antenatal care
AOR: Adjusted odds ratio
CI: Confidence interval
COR: Crude odds ratio
DHS: Demographic and Health Survey
EDHS: Ethiopian Demographic and Health Survey
FMOH: Federal Ministry of Health
FP: Family planning
HSTP: Health Sector Transformation Plan
IUD: Intrauterine device
IPPIUCD: Immediate Postpartum Intrauterine Device
LBW: Low birth weight
MCH: Maternal and child health
MCSP: Maternal and child survival program
MNCH: Maternal, newborn, and child health
NGOs: Non-governmental organizations
PPF: Postpartum family planning
PPIUD: Postpartum intrauterine device
SPSS: Statistical Package for Social Sciences
WHO: World Health Organization
